# Comprehensive Tracheobronchial Morphometry in Korean Adults: Clinical Implications for Double-Lumen Tube Sizing and Right Upper Lobe Alignment

**DOI:** 10.3390/jcm15010318

**Published:** 2026-01-01

**Authors:** Seihee Min, Youn Joung Cho, Jae-Hyon Bahk

**Affiliations:** 1 Department of Anesthesiology and Pain Medicine, Asan Medical Center, University of Ulsan College of Medicine, 88 Olympic-ro 43-gil, Songpa-gu, Seoul 05505, Republic of Korea; 2 Department of Anesthesiology and Pain Medicine, Seoul National University Hospital, Seoul National Univeristy College of Medicine, 101 Daehak-ro, Jongno-gu, Seoul 03080, Republic of Korea

**Keywords:** tracheobronchial anatomy, airway dimension, computed tomography, one-lung ventilation, airway device placement, double-lumen tube

## Abstract

**Background/Objectives:** Accurate knowledge of tracheobronchial anatomy is essential for safe airway management, particularly during one-lung ventilation using double-lumen tubes (DLTs). However, population-specific morphometric data in Asian adults remain limited. We aimed to establish a comprehensive morphometric profile of the tracheobronchial tree in Korean adults using 2D and 3D computed tomography (CT), and evaluate the clinical implications for DLT sizing and right upper lobe (RUL) alignment. **Methods:** This retrospective observational study included 398 adults who underwent preoperative chest CT. Measurements included tracheal dimensions, bronchial lengths, bronchial diameters, and anteroposterior angle of the RUL orifice. Height tertiles and sex-stratified linear regression analyses were performed to evaluate height–bronchial diameter relationships. **Results:** Bronchial dimensions were larger in men; however, height was more closely related to bronchial diameter in women. In women, each 1 cm increase in height corresponded to a 0.071 mm increase in left and a 0.077 mm increase in right bronchial transverse diameter (*p* < 0.001 for both). The RUL orifice showed posterior deviation of 15.5 ± 12.2° in men and 9.9 ± 11.4° in women, with height and weight being independent but weak predictors (R^2^ = 0.05). Bronchial diameter measurements showed consistent differences between 2D and 3D CT, with 2D images generally overestimating transverse diameters. **Conclusions:** The present analysis provides population-specific reference values for Korean adults. Our findings support the use of 2D CT as a practical tool for estimating bronchial dimensions and guiding DLT selection, and may serve as foundation for future airway devices tailored to Asian populations.

## 1. Introduction

An accurate understanding of tracheobronchial anatomy is essential for airway management, particularly during thoracic surgery requiring one-lung ventilation [1,2,3]. The double-lumen tube (DLT) remains the most widely used device for lung isolation, and its performance depends on proper sizing and positioning within the tracheobronchial tree. An undersized DLT may result in insufficient lung separation or intraoperative air leaks, whereas an oversized DLT increases the risk of mucosal injury, bronchial erosion, or postoperative airway complications [4,5,6].

Although several morphometric studies have investigated tracheal and bronchial dimensions, most data were collected from Western or Chinese populations [7,8,9,10]. Anatomical differences among ethnic groups—including tracheal diameter, bronchial length, and airway angulation—are well-known; however, population-specific datasets for Asian adults remain limited [11,12]. Given the increasing global emphasis on personalized airway management and the diversity of available airway devices, establishing an accurate morphometry for specific populations has important clinical implications.

Height is widely recognized as a major predictor of airway size; however, its relationship with bronchial diameter has not been clearly quantified in Korean adults [13,14]. It is unclear whether this relationship is consistent across sexes and whether height-indexed airway models can be reliably used to guide DLT selection [15,16]. Additionally, although inadequate alignment of the ventilating slot with the right upper lobe (RUL) bronchus is a well-recognized cause of lobar obstruction and hypoxemia during right-sided DLT placement, the orientation and angulation of the RUL bronchial orifice have not been systematically characterized in this population.

This study aimed to (1) identify comprehensive tracheobronchial morphometric reference values for Korean adults using 2D and 3D CT, (2) evaluate demographic predictors of airway dimensions, (3) compare 2D and 3D CT measurements to assess the usefulness of routine 2D scans, and (4) analyze the RUL orifice angle with relevance to right-sided DLT placement. By broadening the analytic scope, this study provides a robust population-specific anatomical reference with direct implications for airway management and DLT selection.

## 2. Materials and Methods

This retrospective study was approved by the Institutional Review Board of Seoul National University Hospital (Seoul, Republic of Korea; IRB No. 1706-035-858). The requirement for informed consent was waived due to the use of anonymized clinical data. This study was conducted and reported in accordance with the Strengthening the Reporting of Observational Studies in Epidemiology (STROBE) guidelines.

### 2.1. Study Population

Adults aged ≥18 years who underwent preoperative chest CT between March and October 2016 were screened. The exclusion criteria were as follows: intraluminal lesions in the main bronchus, abnormalities of the tracheobronchial tree, history of tracheobronchial injury or surgery, a diagnosis of musculoskeletal deformities, and poor image quality.

### 2.2. CT Image and Measurement Protocol

Since this was a retrospective study, we collected data from 398 patients who had undergone CT scans using one of the following CT scanners: Sensation 16 (Siemens Medical Solutions, Forchheim, Germany), Somatom Definition (Siemens Medical Solution, Forchheim, Germany), Brilliance 64 (Philips Medical Systems, Best, The Netherlands), or Discovery CT750 HD (GE Medical Systems, Waukenha, WI, USA). All soft-copy images were retrieved from the institutional image archive and communication system, and displayed with bronchial window settings of −450 Hounsfield units and a width of 1000 Hounsfield units [17,18].

**Figure 1:**
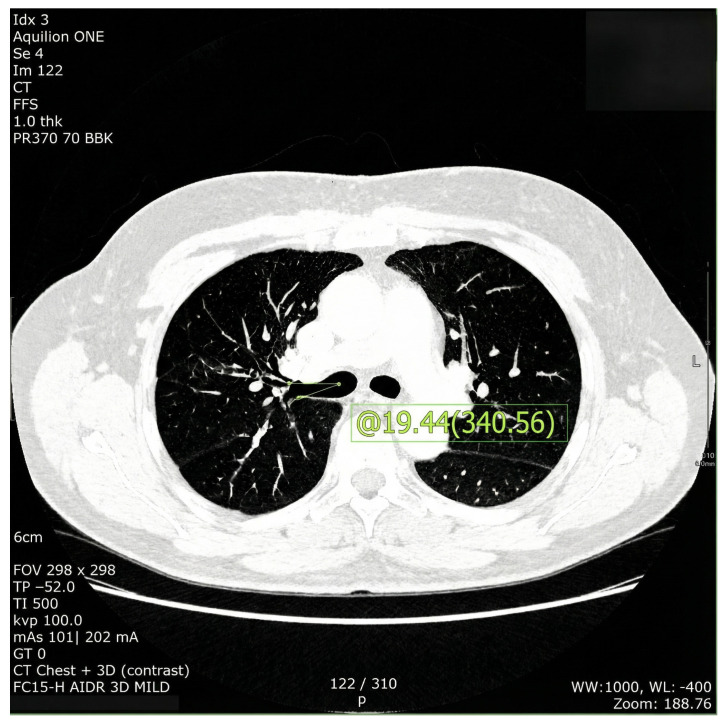
Axial image of chest computed tomography selected for measurement of angle between the horizontal midline of the right main bronchus and the center of the right upper lobe orifice.

Variables were measured by a single investigator (S. M., a thoracic anesthesiologist under the supervision of thoracic radiologists) using electronic calipers on 10x CT images. The position of the RUL orifice was measured from axial 2D CT images using the angle between the horizontal midline of the right main bronchus and the center of the RUL orifice (Figure 1) [19].

Tracheal diameter was measured at the level of the cricoid cartilage and midclavicle. The lengths of the right and left main bronchi (RBL and LBL, respectively) were measured from the tracheal bifurcation to the point where their first branches were located, on both 2D and 3D CT images. The internal diameters of the right and left main bronchi (RBD and LBD, respectively) were measured 1 cm distally from the tracheal bifurcation, since the bronchial cuff of an appropriately positioned DLT has the largest diameter at this position. The diameters were measured anteroposteriorly on axial 2D CT images and mediolaterally on scout images to be compared with the actual diameter perpendicular to its long axis on 3D CT images [20,21,22].

### 2.3. Sample Size Calculation and Statistical Analysis

As this was an exploratory morphometric study aimed at establishing comprehensive reference values, formal a priori sample size calculation was not performed. We included all consecutive eligible patients during the study period to maximize dataset representativeness.

All data are presented as mean ± SD, mean (95% confidence interval [CI]), or number. Independent Student’s *t*-test was used to compare sex-related differences in tracheobronchial dimensions. Sex-specific linear regression was performed for height and bronchial diameters. Prediction equations and 150–180 cm reference tables were generated. Multivariable regression was used to evaluate predictors of the RUL angle. To avoid multicollinearity, the variance inflation factor (VIF) was examined. A *p* value of <0.05 derived from a 2-tailed test was considered statistically significant. SPSS version 23.0 for Windows (SPSS Co., Chicago, IL, USA) was used for the statistical analysis.

## 3. Results

A total of 398 patients were analyzed, including 217 men and 181 women. The clinical and demographic characteristics of the patients are presented in Table 1.

Across all airway parameters, men demonstrated significantly larger tracheal and bronchial dimensions than women. On 3D CT, the mean left main bronchial transverse diameter (LBD_Tr) was 13.15 ± 1.66 mm in men and 10.39 ± 1.30 mm in women, while the right bronchial transverse diameter (RBD_Tr) was 14.25 ± 1.96 mm in men and 12.42 ± 1.62 mm in women. These findings confirm the existence of pronounced sex differences in baseline airway anatomy in the studied population (Table 2). Moreover, 2D CT consistently underestimated bronchial diameters compared with 3D CT, particularly in obliquely oriented bronchi. Despite measurement discrepancies, patterns of sex-based and height-based variation remained robust across modalities (Table 2).

**Figure 2:**
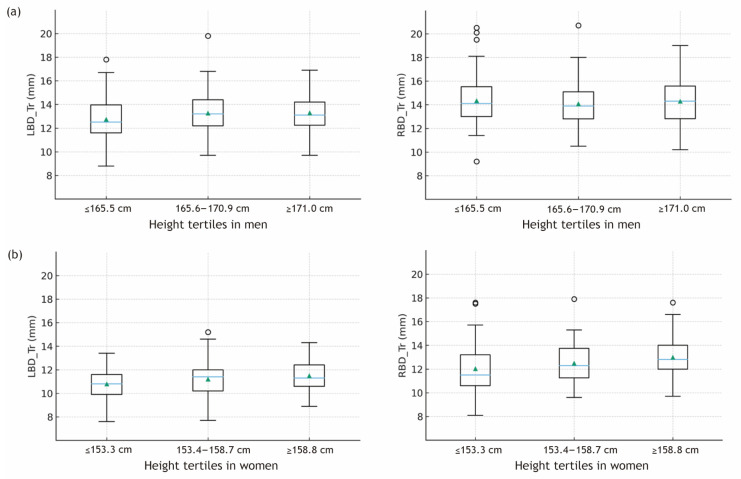
Transverse bronchial diameters across sex-specific height tertiles based on 3D CT measurements. Boxplots show the distribution of left and right main bronchial diameters within each height tertile. Height tertiles were defined separately for (**a**) men (≤165.5 cm, 165.6–170.9 cm, ≥171.0 cm) and (**b**) women (≤153.3 cm, 153.4–158.7 cm, ≥158.8 cm) using sex-specific 33rd and 67th percentiles. Triangle within the box indicates mean value; horizontal line within the box indicates median value; lower and upper boundaries of the box indicate 25th and 75th percentiles, respectively; horizontal lines outside the box indicate 1.5 × 25th and 75th percentiles of the data, respectively. Circles outside the box are outliers. LBD_Tr, transverse left main bronchus diameter; RBD_Tr, transverse right main bronchus diameter.

When patients were stratified by height tertiles, different patterns emerged according to sex. For descriptive comparisons, height was categorized into tertiles separately for men and women using the sex-specific 33rd and 67th percentiles. In men, increase in bronchial diameter across height tertiles were not significant (*p* = 0.093 for LBD_Tr and *p* = 0.678 for RBD_Tr; Figure 2a). In contrast, women exhibited a consistent increase in bronchial diameter across height tertiles. The LBD_Tr increased from 10.81 ± 1.22 mm in the shortest tertile (≤153.3 cm) to 11.50 ± 1.27 mm in the tallest tertile (≥158.8 cm) (*p* = 0.020; Figure 2b). Similar trends were observed in RBD diameters, with RBD_Tr increasing from 12.04 ± 1.94 mm to 13.00 ± 1.57 mm across tertiles (*p* = 0.013; Figure 2b).

**Figure 3:**
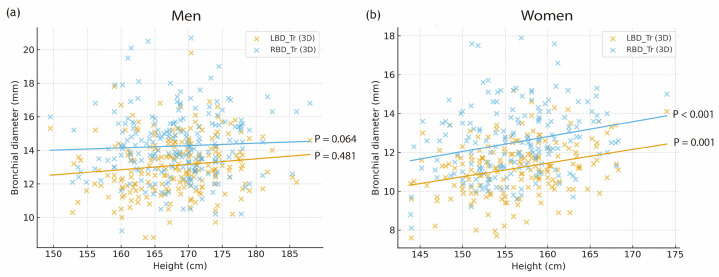
Relationship between height and transverse bronchial diameters measured on 3D CT in (**a**) men and (**b**) women. LBD_Tr, transverse left main bronchus diameter; RBD_Tr, transverse right main bronchus diameter.

In men, linear regression analysis demonstrated only weak and non-significant associations between height and bronchial dimensions (LBD_Tr: β ≈ 0.032 mm/cm, *p* = 0.064; RBD_Tr: β ≈ 0.014 mm/cm, *p* = 0.481; Figure 3a). Although the regression coefficients were positive, their small magnitude and lack of statistical significance indicate that height contributes minimally to bronchial size variation in men. Nevertheless, the consistent directional increases observed across height tertiles supported the development of supplemental height-reference models for use in clinical situations where CT-based measurements are unavailable. In contrast, height was a significant independent predictor of bronchial diameter in women. Each 1-cm increase in height was associated with a 0.071-mm increase in LBD_Tr (95% confidence interval [CI], 0.029–0.114; *p* < 0.001) and a 0.077-mm increase in RBD_Tr (95% CI, 0.031–0.122; *p* = 0.001; Figure 3b).

Height showed no meaningful association with anteroposterior (AP) bronchial dimensions in men, with both LBD_AP and RBD_AP demonstrating negligible and non-significant relationships. In women, height also showed a generally positive but modest association with AP diameters; only the right main bronchial AP diameter demonstrated statistical significance (*p* = 0.009).

Based on these results, height-indexed bronchial prediction equations were generated.

For men, supplemental formulas included:

LBD_Tr ≈ 0.05 × height (cm) − 1.92

RBD_Tr ≈ 0.008 × height (cm) + 12.03

For women, the clinically applicable formulas were:

LBD_Tr = 0.071 × height (cm) − 1.56

RBD_Tr = 0.077 × height (cm) − 2.03

Height-specific reference tables from 150 to 180 cm were constructed using these equations. These regression coefficients were subsequently used to generate height-based predicted bronchial dimensions, which are presented in Table 3.

To further explore the clinical implications of these height-based predictions, predicted bronchial diameters were compared with the distribution of CT-measured values.

In women, substantial overlap was observed across height categories. The shortest tertile (≤153.3 cm) showed mean LBD_Tr of 10.81 ± 1.22 mm, while the tallest tertile (≥158.8 cm) showed 11.50 ± 1.27 mm (*p* = 0.020). Height-based prediction yielded 10.15 mm for a 150-cm woman and 10.86 mm for a 160-cm woman (Table 3). The considerable standard deviations indicate substantial individual variation, suggesting that reliance on height alone may result in selection of DLT bronchial lumen sizes exceeding patient’s actual bronchial diameter in a non-negligible proportion of shorter women.

In contrast, men demonstrated wider dispersion with weak height-diameter correlations (LBD_Tr: β ≈ 0.032 mm/cm, *p* = 0.064; RBD_Tr: β ≈ 0.014 mm/cm, *p* = 0.481). Standard deviations remained substantial across all height tertiles (approximately ± 1.58–1.72 mm), indicating that height-based predictions in men show greater variability and less consistent alignment with measured bronchial diameters, further indicating that height alone may be insufficient to accurately reflect individual airway dimensions.

The RUL orifice angle demonstrated extensive anatomical variability. The mean RUL angle was significantly greater in men than in women, suggesting greater posterior deviation (*p* < 0.001; Table 4). Height and weight showed statistically significant but clinically negligible associations with RUL angle, and the predictive model explained only 5% of the variance (R^2^ = 0.05).

## 4. Discussion

The present study provides one of the most comprehensive analyses to date of tracheobronchial morphology in Korean adults and identifies sex-specific and height-dependent variations that have direct clinical implications for DLT selection. The results demonstrate clear and robust sex-related differences in tracheal diameter, bronchial length, and bronchial diameter. These findings are clinically meaningful because airway dimensions are key factors in the selection of the most appropriate DLT size, prediction of the ease of bronchial intubation, and minimization of airway injury risks [5,23]. DLTs are commonly used for lung separation during surgery and other clinical situations. Thus, both right- and left-sided DLTs can be safely used if they are chosen based on accurate tracheobronchial tree anatomy. Our measurements provide reference values for the right upper lobar bronchial angle to be used in right-sided DLT intubation. The results also highlight the existing variations in the anatomy of the tracheobronchial tree according to patient demographics.

The consistently larger tracheal and bronchial dimensions recorded in men align with previous morphometric findings in both Western and Asian cohorts, confirming that sex is one of the strongest intrinsic determinants of airway anatomy [7,8,13,14]. Because commercial DLTs are manufactured in discrete size categories, accurate understanding of these anatomical differences is essential to reduce the risk of selecting a device of the wrong size. Oversized DLTs may cause mucosal trauma or increased airway resistance, whereas undersized tubes increase the risk of malposition, inadequate lung isolation, and experiencing difficulty in maintaining one-lung ventilation [4,5,10].

The significant sex-specific differences identified in this study emphasize the need for sex-based size selection, particularly in populations such as the Korean, in which smaller airway dimensions than those from Western cohorts are common. Similar sex-related discrepancies were observed in bronchial diameter, which plays a central role in determining the bronchial lumen size of a DLT. The transverse diameter of the left main bronchus, in particular, is a key reference point because left-sided DLTs remain the most commonly used devices for lung isolation. Among the most notable findings in this study was the fact that the close relationship between height and bronchial diameter in women, which contrasts sharply with the weaker one observed in men. In women, an increase of approximately 0.07 to 0.08 mm per centimeter of height may translate to nearly 1 mm across the normal adult height range. Because DLT bronchial lumen sizes increase in 1-mm increments, this variation may directly influence the selection of an appropriate DLT size. Shorter female patients, in particular, exhibit narrower bronchial diameters and may require smaller DLT sizes than those usually recommended based solely on Western anatomical standards. Height-indexed models therefore offer a practical solution for improved DLT selection, especially when CT measurements are unavailable. In contrast, the weak association between height and bronchial diameter in men suggests greater anatomical heterogeneity. While men have larger bronchi overall, the factors governing bronchial size appear to be more complex and less directly tied to height. Variability in chest wall configuration, rib cage morphology, and lung volume may play a more prominent role in determining male bronchial dimensions [24]. Therefore, height-based formulas in men should be used only as supplemental tools rather than as primary determinants of DLT selection.

We also aimed to determine the anatomical position of the right upper lobe orifice by measuring the anteroposterior angle on axial images to provide reference values that may aid right-sided DLT intubation. The RUL bronchial angle exhibited substantial inter-individual variability and a moderate difference between the sexes. We found that the angle of the right upper lobar bronchus is greater in men than in women, and that it has weak but significant linear relationships with height and weight (R^2^ = 0.05). Although this parameter is normally considered during placement of right-sided DLTs, the present findings indicate that demographic factors alone cannot accurately predict RUL orientation [22,25]. Therefore, the RUL angle should be regarded as supplementary information rather than as a primary determinant of airway device selection. Nonetheless, the descriptive data provided in this study may help anesthesiologists anticipate the rotational adjustments required for optimal alignment when a right-sided DLT is clinically indicated. A previous study reported that the incidence of malposition and repositioning of the Mallinckrodt Broncho-Cath™ right-sided DLT was significantly reduced by enlarging the ventilating slot by approximately 100% [26]. This result implies that the use of right-sided DLTs with adequate knowledge of the anatomy of the right upper lobe enables even trainees to perform right-sided DLT intubation safely.

Considering these factors, this study has implications for the design of airway devices. East Asian populations, including that of Korea, have smaller average bronchial diameters than those of Western populations [7,8,10,11,13]. The provision of region-specific morphometric data may inform the design of DLTs better suited to these populations, potentially reducing complications associated with oversized bronchial lumens.

To help with the selection of appropriately sized DLTs, we measured tracheobronchial tree variables on 2D and 3D CT images and compared them with each other. Three-dimensional CT has recently been developed to provide multiplanar reconstruction images by combining 2D images [20,27,28]. Although 3D CT is helpful to determine the accurate length and diameter of the main bronchus for DLT selection, it is expensive and not widely available. Therefore, we compared the measurements obtained using 2D CT and 3D CT data, and evaluated their line of best fit. Although 3D CT appeared to yield slightly greater length values than 2D CT, the magnitude and direction of the sex differences remained consistent across imaging modalities (Table 2). The degree of discrepancy between 2D and 3D CT varied according to the specific anatomical structure. The largest differences were observed in the transverse diameter of the right main bronchus, whereas the smallest ones corresponded to the anteroposterior diameter of the left main bronchus, likely due to obliquity of the bronchi relative to the axial imaging plane [19,27,29]. Despite these discrepancies, the linear relationship between measurements from both modalities remained consistent, suggesting that 2D CT preserves relative differences across patients and may therefore be considered as adequate for clinical decision-making in most preoperative settings. The strength of the relationship also supports previous reports that bronchial diameters measured on axial CT can reliably guide DLT size selection with appropriate caution. Importantly, CT-based measurements should be regarded as an adjunct rather than a replacement for bronchoscopic confirmation. Even with optimal preoperative imaging, real-time bronchoscopy remains indispensable for ensuring correct DLT positioning and preventing lobar obstruction.

This study has several limitations. First, this is a retrospective analysis, and involved multiple CT scanners with varying imaging parameters. However, the large sample size and standardized measurement technique likely mitigated the impact of these factors. Second, all measurements were performed by a single investigator, which ensured internal consistency but limited assessment of inter-observer reliability. Future studies incorporating multiple independent observers would strengthen reproducibility. Third, commercially available DLTs are manufactured in several predefined dimensions, which restricts the individualized selection of DLTs according to measurements from the tracheobronchial tree. However, the results of this study may help clinicians to avoid selecting a DLT of inappropriate size, and suggest the need to increase the variety of size and tip angles available from the manufacturers. Fourth, additional factors such as bronchial length, tracheal shape, and thoracic cavity configuration also contribute to airway anatomy but were not considered in this study. Fifth, although we explored the potential limitations of height-based DLT selection by comparing height-predicted and CT-measured bronchial diameters, this imaging-based study did not include actual DLT size selection or clinical placement outcomes; therefore, the exact rate of incorrect DLT placement could not be quantified. Despite these limitations, the present study provides comprehensive airway morphometry data and height-indexed models that can be used to guide DLT selection. By integrating sex-specific anatomical patterns, height-based predictions, and 3D imaging accuracy, this work contributes to the customization of airway management in Asian patients.

## 5. Conclusions

This study provides detailed morphometric data on the tracheobronchial anatomy of Korean adults, emphasizing the marked differences related to sex and body size that should be taken into account for airway management. These findings provide population-specific descriptive reference data that may inform preoperative assessment and future research on individualized DLT selection, but should be interpreted in conjunction with bronchoscopic confirmation and clinical judgment. The additional information regarding RUL orientation can serve as a useful adjunct for right-sided DLT placement, although this should not replace direct bronchoscopic confirmation.

## Figures and Tables

**Table 1:** Baseline characteristics of study participants.

Variable	Men (n = 217)	Women (n = 181)
Age (years)	56.4 ± 9.8	54.8 ± 8.7
Height (cm)	172.6 ± 6.8 (range, 149.5–188.0)	159.2 ± 5.9 (range, 143.9–174.0)
Weight (kg)	74.3 ± 8.2	59.5 ± 7.1
BMI (kg/m^2^)	24.9 ± 2.3	23.4 ± 2.6

Values are presented as mean ± standard deviation or range. BMI, body mass index.

**Table 2:** Tracheobronchial diameters measured on 2D and 3D CT images.

Anatomical Structure	Sex	Parameter	2D (mm)	3D (mm)	Mean Diff (3D-2D)	*p* Value
Trachea	Men	Tr	19.4 ± 1.5	19.8 ± 1.6	+0.4 ± 0.3	0.002
		AP	21.0 ± 1.7	21.3 ± 1.7	+0.3 ± 0.4	0.041
	Women	Tr	15.9 ± 1.2	16.2 ± 1.3	+0.3 ± 0.3	0.015
		AP	18.1 ± 1.5	18.4 ± 1.5	+0.3 ± 0.4	0.034
LMB	Men	Tr	13.3 ± 1.2	13.6 ± 1.3	+0.3 ± 0.3	0.021
		AP	11.5 ± 1.1	11.8 ± 1.1	+0.3 ± 0.3	0.040
	Women	Tr	11.6 ± 1.2	11.8 ± 1.2	+0.2 ± 0.3	0.066
		AP	10.0 ± 1.0	10.1 ± 1.0	+0.1 ± 0.2	0.182
RMB	Men	Tr	14.7 ± 1.3	15.1 ± 1.4	+0.4 ± 0.4	0.018
		AP	13.0 ± 1.3	13.3 ± 1.3	+0.3 ± 0.3	0.027
	Women	Tr	13.1 ± 1.1	13.4 ± 1.1	+0.3 ± 0.4	0.049
		AP	11.5 ± 1.1	11.7 ± 1.1	+0.2 ± 0.3	0.072

Values are presented as mean ± standard deviation. *p* values were derived from paired *t*-tests comparing 2D and 3D measurements within the same subjects. LMB, left main bronchus; RMB, right main bronchus; Tr, transverse; AP, anteroposterior.

**Table 3:** Mean predicted bronchial diameter according to height.

Height (cm)	Men		Women	
	LBD (cm)	RBD (cm)	LBD (cm)	RBD (cm)
150	13.25	13.75	10.15	10.85
155	13.38	13.90	10.50	11.24
160	13.50	14.06	10.86	11.62
165	13.62	14.21	11.21	12.01
170	13.75	14.37	11.57	12.39
175	13.88	14.52	11.92	12.78
180	14.00	14.68	12.28	13.16

Values are presented as mean. Height tertiles were defined using the sex-specific 33rd and 67th percentiles. LBD, left main bronchus diameter; RBD, right main bronchus diameter.

**Table 4:** Distribution of right upper lobe orifice angle.

Sex	Mean (°)	SD (°)
Men	43.7	11.2
Women	46.5	10.8

Values are presented as mean and standard deviation (SD).

## Data Availability

The data that support the findings of this study are available from the corresponding author upon reasonable request.

## Abbreviations

The following abbreviations are used in this manuscript:

DLT	Double-lumen tube
RUL	Right upper lobe
LBL	Left main bronchi length
RBL	Right main bronchi length
LBD	Left main bronchi diameter
RBD	Right main bronchi diameter
VIF	Variance inflation factor
LBD_Tr	Left bronchial transverse diameter
RBD_Tr	Right bronchial transverse diameter
